# Precision and Bias of Graphite Furnace Analysis of Environmental Samples

**DOI:** 10.6028/jres.093.056

**Published:** 1988-06-01

**Authors:** Gerald L. McKinney

**Affiliations:** U.S. EPA Region VII Laboratory, 25 Funston Road, Kansas City, KS 66115

## 1. Introduction

This study was undertaken to examine the effects of several operational modes of graphite furnace analysis on the precision and bias of environmental trace element analysis. The modes compared were:

1. Method of Standard Additions (MSA) vs Direct Calibration
2. Single vs Double injections
3. Peak Height vs Peak Area Quantitation

The six solid and two water matrices studied are listed in table 1. These were chosen so that results obtained could be compared to a reference value. The elements studied were arsenic, selenium, cadmium, and lead.

## 2. Sample Preparation

The solid matrix samples were prepared using the nitric acid-hydrogen peroxide digestion originally proposed by the EPA EMSL/Cincinnati which is currently included in the EPA contract lab program protocol for low to medium concentration environmental samples.

The aqueous samples were not digested but were spiked to provide 50 *μ*g/L of the elements of interest. Since the purpose of this study was to investigate instrumental precision and accuracy, samples were prepared only once and the four replicate determinations were performed on the same digestate. The precision estimates do not include any variability, due to that of digestion.

## 3. Instrumentation

Analysis was performed using a Perkin-Elmer Zeeman 5000 atomic absorption spectrophotometer equipped with an AS-40 auto-sampler and 3600 data station^1^.

## 4. Analysis Protocol

The study was designed so that all three desired comparisons could be done from a single run by storing each atomization peak on a computer disk. Each sample matrix was injected in duplicate in the following manner:

Cup 1—Straight sample.
Cup 2—Sample diluted 50% with 0.5% HNO_3_.
Cup 3—Sample diluted 50% with 20 *μ*g/L standard.
Cup 4—Sample diluted 50% with 50 *μ*g/L standard.

A computer program calculates peak height and peak area for each atomization peak so that the first injection for cup 1 was used for the direct calibration single injection for both peak height and peak area. The average value obtained from the first and second injections was used for the duplicate injection calculation. A separate least squares linear fit program provided the MSA values from cups 2, 3, and 4. This protocol was repeated on three additional runs, usually on different days.

## 5. Results

All results were normalized to the appropriate percentage of the reference or theoretical value. The concentration in solution in *μ*g/L is also shown. Thirteen of the 32 required dilutions varying from 1–2 to 1–200 to bring them on scale for HGA analysis. Table 2 shows the mean and standard deviation for each comparison.

## 6. Statistical Evaluation

### 6.1 Bias

Comparison of methods for bias (differences between the modes compared) was accomplished by pooling the mean recoveries of each method and using Student’s *t* test in the form:

$$t=\frac{\bar{d}\sqrt{n}}{s_{d}},$$

where

$\bar{d}$: = average difference between each “method mean”
*s*_d_: = standard deviation of these differences
*n*: = number of differences

If *t>t*_.975_, i.e., 3.18 in the case of four replicates, then the means of the two methods are considered to be statistically significantly different.

### 6.2 Precision

Equality of pooled within-sample variance for the modes compared was tested using the *F* test in the form:

$$F=\frac{s_{m_{1}}^{2}}{s_{m_{2}}^{2}},\;\text{where}\;s_{m_{1}}^{2}>s_{m_{2}}^{2}$$

If *F>F*_.975_, i.e., 15.4 in the case of four replicates, then there is a statistically significant difference between variances.

## 7. Discussion

### 7.1 Precision

There were no significant differences in variance for any of the comparisons as summarized in table 3. Precision is not improved with peak area as compared to peak height or method of additions vs direct calibration.

Although not significant in this study possibly due to the small number of replications, we believe generally that precision is degraded in MSA analysis due to the multiple injections required. One of the primary objectives of this work was to investigate the value of duplicate injections. This practice is common in many, if not most, analytical laboratories. Duplicate injections essentially double the analysis time. If precision is not improved, then single injections would improve a laboratory’s efficiency. This study indicated no significant improvement in precision. It has been our experience that poor precision is usually due to what we call “correctable problems.” These include poor injection practices; improper furnace conditions; tube, platform, or contract ring conditions; inadequate background correction; or improper matrix modification. These and other such conditions should be corrected before continuing analysis. Duplicate injections do not correct these conditions. As a result of this work and discussions with other analysts, we have discontinued the practice of duplicate injections.

### 7.2 Bias

In the context of this study, bias is concerned with the agreement of the two modes compared. As was anticipated, direct calibration produced results that were generally lower than those by the method of additions. We have observed that interferences do sometimes tend to bias direct calibration results low. We have also noticed a high bias in the MSA results which we have not been able to explain.

For Se and Cd, the differences between direct calibration and MSA were not statistically significant. The 15% difference found for arsenic was significant. Both direct calibration and MSA gave only 26% recovery for sample type 5—the EPA Municipal Digested Sludge sample. We believe, as do other workers, that the true value may be less than that found by analysis [1]. Looking at the other seven sample types, the MSA mean recovery is 106% and the direct calibration is 88%, indicating that even though the MSA value is biased high, it is somewhat better than that resulting from direct calibration. The statistically significant difference found for lead is interesting in that the direct calibration results were somewhat better than the MSA results. The direct calibration averaged 96% recovery, and the MSA averaged 108%. Lead is a good example of the strides that have been made in eliminating interferences in graphite furnace. Only a few years ago, lead recoveries were often low. This was usually attributed to chloride interferences. Now accurate results may be obtained for lead even in hydrochloric acid.

Selenium analysis is a good example of how statistical evaluation may sometimes be misleading. The differences in precision and bias found between MSA and direct calibration were not statistically significant. However, the difference for the NBS oyster tissue was the largest of any observed, the recoveries being 86% for MSA and only 41% by direct calibration. Since selenium is often present in tissue at significant levels, accurate analysis is important. These results indicate that one would not want to attempt selenium analysis for tissue using these conditions by direct calibration. This example demonstrates that, for complex and difficult matrices, more work needs to be done to remove interferences. But just as this is evident, we also believe that it is possible to overcome these interferences. For selenium and arsenic, perhaps palladium or a mixed palladium matrix modification may be the answer (see [2]). Although there are cases such as the above where the method of additions is required, we feel that there are disadvantages other than the time required that are often overlooked and hence we avoid MSAs whenever possible. These include errors when the sample to spike ratio is inappropriate, or small errors in calibration and blank (baseline) that can be magnified in MSAs. The sum of the sample and spike can put analysis out of the linear range. Small variations in the individual readings in an MSA determination can produce larger errors in the final result. One example which is quite typical from this study demonstrates this last problem. In the MSA value for arsenic in orchard leaves, the fourth replicate was biased positive 49%. Since the correlation coefficient of calibration was low, this replicate was repeated. The individual readings of the second run were very close to the first run with the largest difference for the highest spike level. The difference between the first and second run for this was 7.7% (33.8 to 36.5 *μ*g/L). In addition, the correlation coefficient improved and the bias was positive (only 15%). That is, a difference of 7.7% in one of the individual readings made a difference of 34% in the final result. This type of error is so common that we prefer to use direct calibration for the most accurate results for routine work such as verifying maximum contamination levels in drinking water or analyzing performance evaluation samples.

Errors introduced due to inappropriate spiking level and calibration error is discussed by Gaind and Odell [3]. They address the EPA contract lab “continuing calibration” criteria of ±10% and its effect on the probability of successful spike recovery. This probability is low and they believe the 10% limit is too wide.

## 8. Conclusion

We believe that the data from this study demonstrate that reliable analytical data can be achieved, whether these data are obtained from peak height or peak area, single or double injections, or direct calibration or the method of additions. Certainly, there are exceptions to every rule, but we believe that advances have made the interferences the exception. These advances include such things as improved background correction, delayed atomization, automatic injection, and matrix modification. We are approaching the “standardless graphite furnace analysis” proposed by Walter Slavin and co-workers [4]. *In* the absence of interferences, absolute calibration by a “characteristic mass” could be a powerful concept in accurate trace analysis.

## Figures and Tables

**Table 1:** Sample types

1.	NBS Spinach
2.	NBS Orchard Leaves
3.	NBS Oyster Tissue
4.	NBS River Sediment
5.	EPA Sludge
6.	Urban Particulate
7.	Drinking Water
8.	Wastewater

**Table 2:** Mean and standard deviation for comparison.

Comparison	Element: Arsenic Sample type								Element: Selenium Sample type							
	1	2	3	4	5	6	7	8	1	2	3	4	5	6	7	8
Method of additions	121	97	102	88	26	116	111	105		15	86			110	107	109
Standard deviation	23	16	17	13	12	7	9	13		8	12			11	14	15
Direct calibration	92	82	81	67	26	97	100	97		23	41			88	97	95
Standard deviation	23	15	12	5	10	14	6	9		14	15			14	11	10
Single injection	92	82	81	67	25	97	100	97		23	41			88	97	95
Standard deviation	23	15	12	5	10	14	6	9		14	15			14	11	10
Double injection	111	78	79	67	27	97	99	94		27	37			105	92	96
Standard deviation	18	5	10	5	10	13	5	13		22	14			9	11	14
Peak height	72	71	64	64	16	88	89	77		19	33			70	98	108
Standard deviation	21	17	11	11	8	14	9	6		10	17			2	10	3
Peak area	92	82	81	67	25	97	100	97		23	41			88	97	95
Standard deviation	23	15	12	5	10	15	6	9		14	15			14	11	10

Comparison	Element: Cadmium Sample type								Element: Lead Sample type							
	1	2	3	4	5	6	7	8	1	2	3	4	5	6	7	8
Method of addition	97	78	110	101	103	109	101	118	111	110	95	110	108	111	110	109
Standard deviation	22	10	4	8	15	7	20	8	22	8	14	6	16	10	3	4
Direct calibration	105	99	98	84	99	103	107	101	100	87	104	95	102	92	99	91
Standard deviation	10	17	6	9	5	7	9	5	16	4	8	12	6	3	6	7
Single injection	105	99	98	84	99	103	107	101	100	87	104	95	101	92	99	91
Standard deviation	10	17	6	9	5	7	9	5	16	4	8	12	5	3	5	7
Double injection	106	132	99	84	100	110	112	112	110	86	98	97	100	92	99	94
Standard deviation	13	8	6	5	4	11	10	14	12	4	11	10	6	4	5	5
Peak height	93	102	82	65	66	101	88	91	82	93	113	94	99	91	108	73
Standard deviation	17	20	23	12	11	5	13	17	8	17	9	10	9	3	9	7
Peak area	105	99	98	84	99	103	107	101	100	87	104	95	101	92	99	91
Standard deviation	10	17	6	9	5	7	9	5	16	4	8	12	5	3	5	7

**Table 3:** Statistical evaluation

Precision—*F* Test				
Comparison	As	Se	Cd	Ph
MSA vs DC	1.3	1.1	2.0	1.9
Pk Ht vs Pk A	1.0	1.7	2.9	1.3
Single vs Double	1.4	1.3	1.1	1.2

Agreement—Student’s *t*				
Comparison	As	Se	Cd	Pb
MSA vs DC	4.7^a^	1.9	0.5	3.2^a^
Pk Ht vs Pk A	4.8^a^	2.4	3.4^a^	0.5
Single vs Double	0.5	0.6	1.8	0.6

No statistically significant differences.

a Indicates statistically significant difference.

## Footnotes

1 Disclaimer: Mention of trade names does not constitute endorsement by U.S. EPA.

## References

[1] Adelman H, Jeniss SW, Katz SA (1981). Amer Lab.

[2] Xiao-quan Shan, Zhe-ming Ni, Li Zhang (1984). Atomic Spectrosc.

[3] Gaind A, Odell G (1987). Consequence of Analytical Spiking Protocol and Continuing Calibration Specifications.

[4] Carnick Glen, Manning David, Slavin Walter (1984). The Possibility of Standardless Graphite Furnace Analysis.

